# Chronic myeloid leukemia with two rare fusion gene transcripts of atypical *BCR::ABL*: A case report and literature review

**DOI:** 10.1097/MD.0000000000036728

**Published:** 2024-01-19

**Authors:** Yuxin Li, Yilin Zhang, Xin Meng, Sheping Chen, Ting Wang, Longjin Zhang, Xiaorong Ma

**Affiliations:** a Department of Hematology, The Second Affiliated Hospital of Xi’an Jiaotong University, Xi’an, Shaanxi, China.

**Keywords:** *BCR: : ABL*, case report, chronic myelogenous leukemia, imatinib, karyotype analysis, literature review

## Abstract

**Rationale:**

Imatinib is a standard treatment for Philadelphia (Ph) chromosome-positive chronic myeloid leukemia (CML), but its efficacy in rare *BCR::ABL* variants is underexplored.

**Patient concerns:**

A 67-year-old woman was admitted to the Second Affiliated Hospital of Xi’an Jiaotong University in March 2022 due to elevated white blood cells.

**Diagnosis:**

Karyotype analysis revealed clonal abnormalities involving the variant t(9;22) and positive results for atypical *BCR::ABL* variants (e14a3 and e13a3). The clinical diagnosis was CML, chronic phase, Ph+, with rare *BCR::ABL*-e13a3- and *BCR::ABL*-e14a3-positive findings.

**Intervention:**

The patient was administered daily imatinib mesylate (400 mg).

**Outcomes:**

After 4 weeks, a swift molecular response was observed: *BCR::ABL*-e13a3 transcript level at 2.82 × 10^−1^ (28.24%), and *BCR::ABL*-e14a3 transcript level at 4.68 × 10^−1^ (46.76%). Within 3 months, a complete cytogenetic response was achieved, with a Ph chromosome ratio of 0. Early molecular response was evident as *BCR::ABL*-e13a3 transcript level reached 5.11 × 10^−3^ (0.51%), and *BCR::ABL*-e14a3 transcript level at 6.26 × 10^−3^ (0.63%). The imatinib mesylate treatment continued without significant toxicity.

**Lessons:**

This case emphasizes the potential effectiveness of imatinib mesylate in managing rare *BCR::ABL* fusion gene variants of CML. Screening for these atypical variants is advised for suspected CML patients who test negative for common *BCR::ABL* fusion gene variants. The presented case underscores the positive outcomes achieved with imatinib treatment for a patient with rare *BCR::ABL* variants, contributing valuable insights for the management of similar cases. Screening for unusual fusion gene variants should be a consideration in CML diagnosis for comprehensive treatment strategies.

## 1. Introduction

Chronic myeloid leukemia (CML) is a malignant tumor formed by the clonal proliferation of bone marrow hematopoietic stem cells, accounting for 15% of adult leukemias.^[1]^ The annual age-adjusted incidence of CML is about 1 to 2 per 100,000 persons/year.^[1–3]^ Patients in the blast phase generally have a poor prognosis, with reported overall survival of < 1 year.^[3]^ The most important prognostic factors are age, spleen size, and myeloblast, platelet, eosinophil, and basophil counts.^[1–3]^

More than 90% of CML occurrences are associated with a translocation between (9;22) (q34; q11) to form the Philadelphia (Ph) chromosome and the *BCR::ABL* fusion gene at the molecular level. This fusion gene is the molecular basis for the occurrence of CML.^[4]^ Differences in the *BCR* or *ABL* gene breakpoints lead to the diversity of *BCR::ABL* fusion genes and clinical features. At present, *BCR::ABL* is divided into 3 types according to the different breakpoints of *BCR* or *ABL*, namely M-type, m-type, and u-type. The M-type includes e13a2 and e14a2 and expresses the p210 protein; the m-type includes e1a2 and expresses the p190 protein; the u-type includes e19a2 and expresses the p230 protein.^[5]^ In addition to the common breakpoints, there are a small number of uncommon breakpoints, called atypical *BCR::ABL* fusion genes.^[6]^ Currently, the main ones are e1a3, e13a3, e14a3, e19a3, e6a2, and e8a2, and these atypical fusion transcripts only appear in 1% to 2% of CML patients.^[7]^ Among them, the atypical *BCR::ABL* fusion gene single a3 type has appeared many times in literature reports in recent years, but CML with 2 atypical e13a3 and e14a3 *BCR::ABL* fusion gene subtypes has not been reported in China.

Patients with newly diagnosed Ph chromosome-positive CML in the chronic phase with adequate tyrosine kinase inhibitor (TKI) therapy typically have a similar prognosis to the general population without CML.^[3]^ Imatinib is a TKI designed to inhibit the *BCR::ABL* protein specifically.^[8]^ Imatinib is a treatment of choice for patients with Ph + CML, and the strongest response is observed with the e14a2 variant,^[1]^ but the data about the response to imatinib in the presence of other rare variants are limited.^[9,10]^ Therefore, this report summarizes the clinical and laboratory characteristics of a patient with CML expressing both e13a3 and e14a3 transcripts and evaluates the clinical efficacy of imatinib mesylate.

## 2. Case report

A 67-year-old female was admitted to the Second Affiliated Hospital of Xi ‘an Jiaotong University on March, 2022, due to elevated white blood cell count (WBC) for 1 week. There was no anemia, no bleeding spots on the skin, no tenderness on the sternum, and no swelling of the superficial lymph nodes. The oral mucosa was smooth without ulceration, and the pharynx had no bleeding. A cardiopulmonary physical examination showed no obvious abnormalities. The spleen was located 3 cm below the ribs of the left midclavicular line, with a medium hard texture and no tenderness. The blood routine analysis showed elevated WBC (93.42 × 10^9^/L), red blood cell count (3.21 × 10^12^/L), hemoglobin (115 g/L), and platelet count (PLT) (339 × 10^9^/L), the neutrophil percentage elevated by 76.7%, and neutrophil count showed 71.54 × 10^9^/L, with basophil count at 10.76 × 10^9^/L.

The bone marrow aspirate revealed active bone marrow proliferation. Granulocytic lineage accounted for 91.00%, erythroid lineage accounted for 4.00%, and granulocytic: red = 22.75:1. The proliferation of the granulocyte system was active, mainly neutrophils, and the proportion of eosinophilic and basophilic cells increased. The proliferation of the erythroid and lymphoid systems was reduced and 3 megakaryocyte systems and platelets can be found.

The bone marrow biopsy showed that the proliferation of bone marrow hematopoietic tissue was significantly active, the proportion of granulocytes was significantly increased, eosinophils were scattered and easily seen, erythroid hyperplasia was reduced, megakaryocytes were slightly less, and fibrotic tissue in the interstitium was significant. CML with myelofibrosis could not be ruled out.

**Figure 1.**
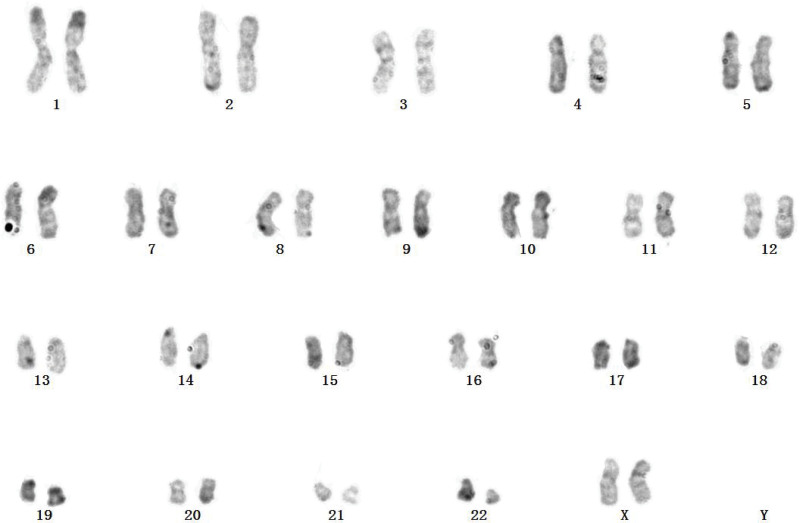
Analysis of the patient chromosomal karyotype.

The flow cytometry results showed that 0.44% myeloid blasts (occupying nuclear cells) were detected in the submitted specimens, and the phenotype was not abnormal; 70.13% granulocytes (occupying nuclear cells) were detected, the relative proportion was increased, and the expressions of CD16 and CD13 were decreased, the development pattern of CD16/CD13/CD11b was abnormal; 7.85% basophils (occupying nuclear cells) were detected, and the proportion was increased; 3.87% eosinophils (occupying nuclear cells) were detected; lymphocytes occupied 1.38% of nucleated cells, and the proportion was significantly reduced. The chromosomal karyotype was 46, XX, t(9;22), as shown in Figure 1.

Real-time quantitative PCR (qRT-PCR) detection showed that the *JAK2, CALR*, and *MPL* genes were negative. In addition, the *BCR::ABL* (p210, p190, p230) genes were negative. In order to determine the *BCR::ABL* breakpoint in this patient, RT-PCR was performed using additional primer sets, including forwarding primers for *BCR* exon 1 (e1), *BCR* exon 12 (e12), and *ABL* reverse primer for exon 3 (a3). These primer sets cover most of the previously reported uncommon breakpoints. *BCR::ABL* rare genotype test results were positive for *BCR::ABL*-e13a3 and *BCR::ABL*-e14a3. The ratio of *BCR::ABL*-e13a3 fusion gene/internal reference gene was 1.37 × 100 (137.22%), and the ratio of *BCR::ABL*-e14a3 fusion gene/internal reference gene was 1.83 × 100 (182.55%).

Finally, the clinical diagnosis was CML, chronic phase, Ph+, rare *BCR::ABL*-e13a3- and *BCR::ABL*-e14a3-positive). The Sokal score was an intermediate risk, and the EUTOS score was a low risk. After diagnosis, the first-generation TKI imatinib mesylate 400 mg/d targeted therapy was given. After 1 month after treatment, the blood routine showed WBC at 2.51 × 10^9^/L, red blood cell count at 3.08 × 10^12^/L, hemoglobin at 107 g/L, PLT at 71 × 10^9^/L, and the percentage of basophils was 4%, indicating that complete hematologic remission was achieved. The *BCR::ABL* transcript level of the *BCR::ABL*-e13a3 fusion gene/internal reference gene ratio was 2.82 × 10^−1^ (28.24%), indicating very early molecular remission (EMR). The *BCR::ABL*-e14a3 fusion gene/internal reference gene ratio was 4.68 × 10^−1^ (46.76%), not reaching very EMR.

Chromosomal karyotype analysis was repeated 3 months after treatment, and the proportion of the Ph chromosome was 0, and the patient achieved complete genetic remission. The *BCR::ABL*-e13a3 fusion gene/internal reference gene ratio was 5.11 × 10^−3^ (0.51%). The *BCR::ABL*-e14a3 fusion gene/internal reference gene ratio was 6.26 × 10^−3^ (0.63%). The response to imatinib was good and achieved EMR.

Up to now, the patient has been treated with TKI for 3 months. During treatment, the drug was temporarily suspended for 3 weeks due to hematological adverse reactions (thrombocytopenia). After the PLT recovered, the drug was taken again at the initial dose. At present, the treatment with imatinib mesylate 400 mg/d is continued, and the treatment effect and adverse drug reactions are regularly evaluated in the outpatient department. The patient tolerated it well, and there were no obvious hematological and non-hematological adverse reactions.

## 3. Discussion

This report summarized the clinical and laboratory characteristics of a patient with CML expressing both e13a3 and e14a3 transcripts and evaluated imatinib mesylate clinical efficacy. Therefore, for patients with clinically highly suspected CML, it is necessary to screen for atypical *BCR::ABL* fusion genes in patients negative for common *BCR::ABL* fusion variants to gain a better understanding of the treatment of rare *BCR::ABL* fusion variants.

CML is characterized by the presence of the Ph and the associated *BCR::ABL* fusion gene. Different transcripts are produced depending on the 2 involved genes’ breakpoints. In CML, most *BCR::ABL* breakpoints occur downstream of exon 13 (e13) or 14 (e14) of BCR and upstream of exon 2 (a2) of *ABL1*. Fusions of *BCR* to *ABL1* exon a3 (e13a3/e14a3) are extremely rare, found in only 0.9 percent of *BCR::ABL1*-positive patients.^[11]^ Structurally, in the case reported here, the breakpoint of the a3 transcript is located downstream of exon a2. Therefore, exon a2 was deleted, and the transcript lacked part of the *ABL* SH3 domain, which is thought to promote leukemia by negatively regulating the kinase domain (SH1) and activating the STAT5 signaling pathway.^[12]^ Therefore, patients with CML who lack part of the A3-type transcript of ABLSH3 might have a better prognosis.^[13]^ In addition, due to the changes in tertiary structure, CML patients with a3-type *BCR::ABL* transcripts might respond differently to TKIs than a2-type patients.^[14,15]^ In the case report, the patient showed a good response to imatinib, with complete hematologic remission after 1 month and EMR after 3 months. Still, only 1 patient and no a2-type patients were examined in the present study, and no conclusion can be drawn regarding prognosis.

In 1991, van der Plas et al^[16]^ first detected transcripts lacking exon a2 by a polymerase chain reaction and Southern blot analysis. In 2003, Liu et al^[17]^ first detected the exact DNA breakpoint of a3 *BCR::ABL* by nucleotide sequencing and reported in detail the diagnosis and treatment of a case of e13a3. They suggested that patients with CML with a3 transcripts show a low proportion of circulating immature cells, mild or no splenomegaly, slow progression, resistance to IFN-α, and a good response to imatinib mesylate.^[17]^ In 2018, Qin et al^[18]^ screened 83 patients with rare *BCR::ABL* transcripts from 4750 CML patients, including 11 cases of e14a3 and 6 cases of e13a3. Compared with CML patients with typical *BCR::ABL* (P210), patients with the a3 type had a better response to imatinib.^[18]^ In 2019, Xue et al^[19]^ identified 40 patients (1.7%) with rare *BCR::ABL1* transcripts from a cohort of 2331 patients with CML, including 7 with e14a3 and 2 with e13a3. The results were the same as in previous studies, i.e., most patients with e13a3/e14a3 transcripts had a better response to TKI than patients with typical transcripts. In 2021, Schafer et al^[20]^ analyzed 33 patients with CML, including 4 patients with e13a3, 6 patients with e14a3, and 2 patients with double transcripts e13a3/e14a3. Almost all patients (83%) achieved deep molecular remission after TKI, and the *BCR::ABL1* value was lower than the detection limit.^[20]^

In the case report, the routine RT-PCR test using a conventional commercial kit was negative. In the subsequent repeated testing and verification by the PCR kit made by our laboratory, the patient *BCR::ABL* was a double transcript e13a3/e14a3. Cases of co-expression of both genotypes have also been reported previously.^[20]^ In 2018, a patient with a double expression of 2 rare types of e18a2 and e19a2 was reported in the Chinese literature, but the treatment response was not reported. By reviewing this case and related literature, it could be found that the simultaneous expression of 2 atypical breakpoints in the *BCR::ABL* fusion gene is very rare in 1 patient, and its biological and clinical effects are still unclear. Nevertheless, most patients with type a3 CML responded well to imatinib mesylate. Considering the age, general condition, Sokal score, and EUTOS score of the patient in this report, imatinib mesylate was also selected for treatment. Although qRT-PCR is the standard test for detecting *BCR::ABL* of CML, it has limits in the breakpoints detected based on the location of the primers and probes.^[21]^ Therefore, further sanger sequencing and FISH were needed in order to verify our result. In addition, whether the patient expressing 2 a3-type transcripts at the same time had a better effect from TKI than other types of transcript requires larger sample size for further verification.

In conclusion, for patients with a high clinical suspicion of CML, it is necessary to re-screen for atypical *BCR::ABL* fusion genes in patients who are negative by conventional tests. Although the available literature suggests that a3-type *BCR::ABL* fusion genes have a good prognosis and response to TKI, more data are necessary to determine the clinical significance of such transcripts. It would facilitate the timely initiation of treatment for a clear diagnosis of the disease, but also helps to reasonably evaluate the prognosis and disease progression of patients so that clinicians can formulate first-line strategies, rationally use drugs, and accumulate practical treatment experience for CML with rare *BCR::ABL* fusion genes.

## Author contributions

**Conceptualization:** Xiaorong Ma.

**Data curation:** Yuxin Li.

**Formal analysis:** Yuxin Li, Yilin Zhang, Xin Meng, Xiaorong Ma.

**Methodology:** Sheping Chen, Ting Wang, Longjin Zhang.

**Writing – original draft:** Xiaorong Ma.

**Writing – review & editing:** Xiaorong Ma.
